# Investigation of the Compressive Viscoelastic Properties of Brain Tissue Under Time and Frequency Dependent Loading Conditions

**DOI:** 10.1007/s10439-021-02866-0

**Published:** 2021-10-04

**Authors:** Weiqi Li, Duncan E. T. Shepherd, Daniel M. Espino

**Affiliations:** Department of Mechanical Engineering, University of Birmingham, Birmingham, B15 2TT UK

**Keywords:** Brain tissue, Finite element, Loss, Model, Modulus, Storage, Validation, Viscoelasticity

## Abstract

The mechanical characterization of brain tissue has been generally analyzed in the frequency and time domain. It is crucial to understand the mechanics of the brain under realistic, dynamic conditions and convert it to enable mathematical modelling in a time domain. In this study, the compressive viscoelastic properties of brain tissue were investigated under time and frequency domains with the same physical conditions and the theory of viscoelasticity was applied to estimate the prediction of viscoelastic response in the time domain based on frequency-dependent mechanical moduli through Finite Element models. Storage and loss modulus were obtained from white and grey matter, of bovine brains, using dynamic mechanical analysis and time domain material functions were derived based on a Prony series representation. The material models were evaluated using brain testing data from stress relaxation and hysteresis in the time dependent analysis. The Finite Element models were able to represent the trend of viscoelastic characterization of brain tissue under both testing domains. The outcomes of this study contribute to a better understanding of brain tissue mechanical behaviour and demonstrate the feasibility of deriving time-domain viscoelastic parameters from frequency-dependent compressive data for biological tissue, as validated by comparing experimental tests with computational simulations.

## Introduction

Brain tissue is soft and complex, and its mechanical characterization has been studied for decades. A recent study showed that the mechanical environment is an essential consideration for neurodevelopment.^6^ Computational simulations are vital for medical training and the design of clinical tools. Also, mechanical models have been proven to be promising methods to analyze the mechanisms of brain injuries and predict the response of brain in various impact conditions.^35, 44^ Finite element (FE) simulations can incorporate real physical loading conditions allowing tissue characterization modelled more accurately and the application of brain tissue in head computational simulations have been recently performed to analyze brain-related injuries.^21, 30–32^ However, the level of accuracy in modelling tissue response requires quantitative data from experiments and depends on the material models. To simulate biological tissues, the simplest model which best describes the mechanical behavior of the tissue is preferred, which can be solved across a variety of loading conditions.^25^

The mechanical behavior of brain tissue has been studied under various test conditions. Brain injuries may be induced by angular, shear and translational force. Oscillations of the head leading to brain shaking within the skull can also produce brain trauma.^26^ Comparison of the mechanical properties of brain tissue in the literature shows that there is a lack of standard testing protocols.^12^ Some studies investigated brain tissue in the time domain^50^ while dynamic sweep tests on brain tissue in the frequency domain have also been performed.^16^ Further, the mechanical results also depend on sample preparation, indenter geometry and measurement length-scale. Compressive loading can lead to brain trauma^1, 54^ and compressive waves were found on the impact site of brain tissue during the course of head dynamics.^36^ Although a range of dynamic mechanical data are available for various materials in the literature, it has rarely been applied in modelling to analyze and design structures, mainly because models are often solved under steady state conditions. Therefore, it is of great practical use to determine time-dependent material properties from frequency-dependent data obtained from mechanical testing.

Brains are viscoelastic and for viscoelastic materials, the relationship between stress and strain is dependent on time. Linear viscoelastic models are commonly used for biological tissue and it has the benefits of being easily optimized in the computation attributed to its physical theory of mechanical models dealing with linear springs and dashpots.^38^ The Prony series was applied in this study since it has been widely used and proved to effectively represent the equations of the material’s viscoelastic properties.^18^ Generally, viscoelastic characterization can be implemented either in the time or the frequency domain and this model is capable of describing the mechanical properties of a material from both testing domains. Based on the equivalent mathematical equations including integral and differential theory with shared linear viscoelastic material parameters, it should be possible to link between time dependent and frequency dependent viscoelastic properties.^47^ Even though frequency dependent properties and corresponding viscoelastic models of brain tissue have been recently studied,^27^ it remains unclear whether such data can be used in computational models to predict mechanical behavior under various loading conditions such as under time-dependent loading. In order to understand the mechanical properties of brain tissue under different testing impact conditions, the frequency-dependent and time-dependent relaxation behavior of brains were studied with the same physical conditions through compressive mechanical testing.

For viscoelastic materials, a dynamic modulus is defined as the ratio of complex stress to complex strain during oscillation with a phase lag between the two waves; whereas, stress relaxation is the temporal response of a material to a constant strain. Dynamic mechanical analysis (DMA) has been considered as an effective technique for measuring the bulk mechanical properties of viscoelastic materials.^4^ This method is flexible and powerful to map frequency-dependent viscoelastic properties of biological tissue over a range of frequencies covering physiological and injury loading conditions. The storage modulus in viscoelastic materials characterizes the ability of the material to store energy in the elastic phase and the loss modulus characterizes the ability of the material to dissipate energy, for instance as heat, in the viscous phase. The relaxation modulus can be determined in the time domain, however, it is limited to the strain rate range used in experiments and it can be time consuming leading to long measurement trials.^55^ Thus, it is of value to characterize viscoelastic properties, such as Prony series, from dynamic moduli which can be used to predict time-domain phenomena such as stress relaxation when applied to FE models.

The purpose of this study was to transform viscoelastic properties obtained experimentally *via* dynamic mechanical analysis to a Prony series, for white and grey brain matter. Prony series parameters were determined using a constitutive model and implemented in FE analysis. The FE model has been evaluated in both time and frequency domains against relevant experimental data.

## Materials and Methods

### Sample Preparation

**Figure 1:**
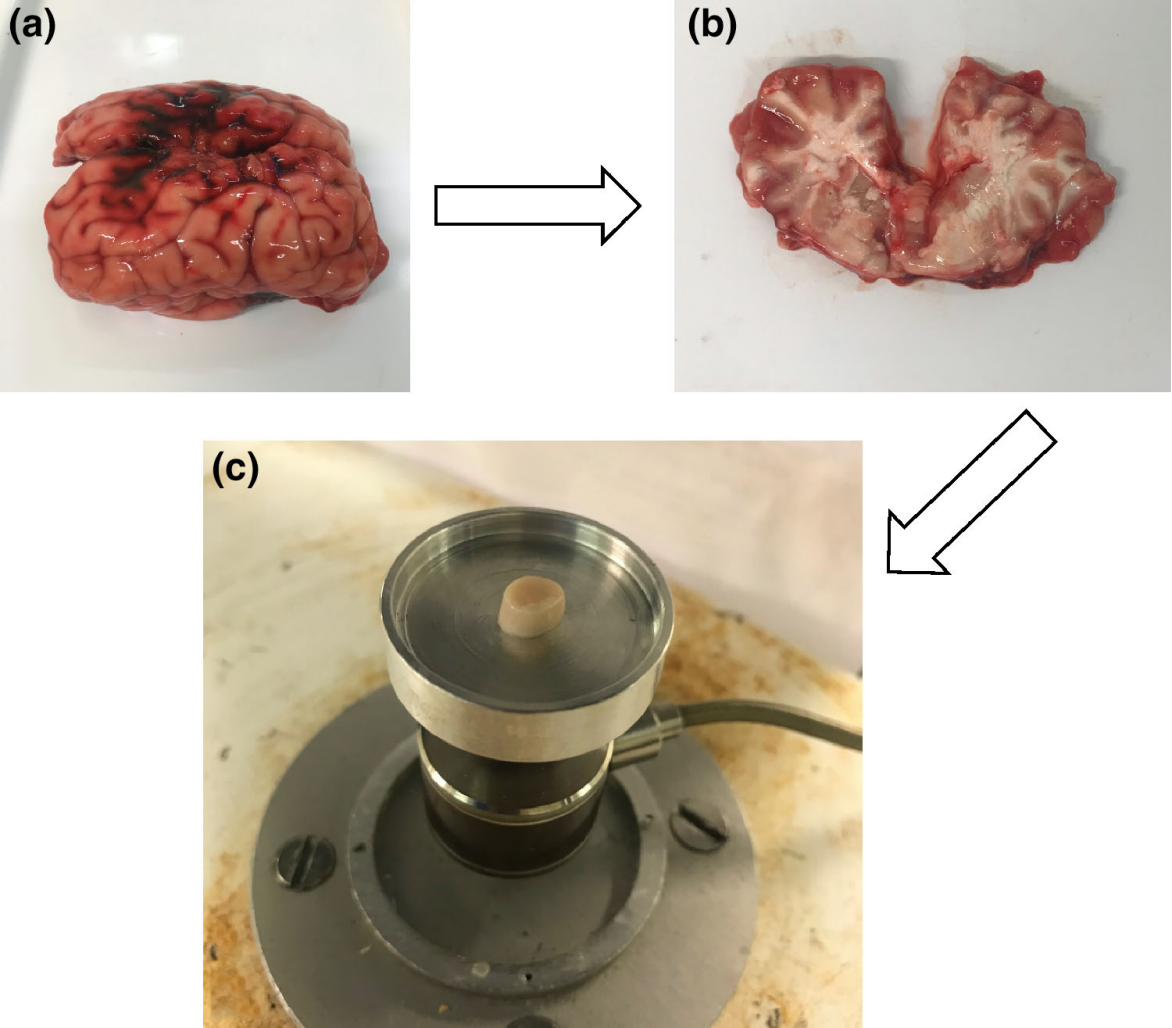
(a) A bovine brain obtained for dissection and specimens were collected from (b) a slice of cerebrum. (c) Representative cylindrical specimen for compressive mechanical testing.

Eight whole bovine brains were obtained from animals under 12 months of age collected from a supplier (Samples for Schools, Portsmouth, UK), and all of the specimens were free from imperfections.^27^ On arrival in the laboratory, the brains were stored at – 40 °C wrapped in tissue paper soaked in Ringer’ solution (Oxoid Ltd, Basingstoke, UK) following the standard procedure.^28, 52^ Prior to the mechanical tests, brain samples were thawed in Ringer’ solution for 12 h before dissection. The freeze-thaw process has not been found to adversely affect the mechanical properties of biological tissue.^11, 43^ Slices of cerebrum were collected from brain tissue using a surgical scalpel (Swann-Morton Limited, Sheffield, UK). During the dissection, specimens were immersed in Ringer’s solution and a circular trephine of 8 mm diameter was applied to extract white and grey matter samples (Fig. 1). The specimens of white matter were collected from regions of the corona radiata and corpus callosum, and the specimens of grey matter were collected from regions of the cortex and basal ganglia, which is in agreement to previous studies.^9, 29^ The variability of measured dimension may be increased due to the soft nature of brain tissue which can cause deformation under its own weight in preparation. Prior to the mechanical tests, geometric dimensions were determined using a Vernier calliper (Draper Tools Ltd, Hampshire, UK). The brain samples obtained were 8 ± 0.1 mm in diameter and 5 ± 0.5 mm (mean ± standard deviation) in thickness.

### Experimental Setup

Mechanical testing was conducted using a Bose ElectroForce 3200 (Bose Corporation, ElectroForce Systems Group, Minnesota, USA) testing machine. This approach has been previously used to test many biological and synthetical materials.^2, 4, 23^ The brain specimens were placed in the sample container; force and displacement values were adjusted to be a zero. Prior to the data collection procedure, an upper flat indenter was lowered onto the specimen until a preload of 10 mN was observed using the WinTest DMA software (Bose Corporation, ElectroForce Systems Group, Minnesota, USA).

The viscoelastic characterization was investigated both in the time (using stress-relaxation) and the frequency domain (using DMA). For DMA, amplitude sweep tests were conducted at 1 Hz to determine the amplitude range within the linear viscoelastic region of the material. Samples were subjected to a pre-strain with a mean displacement of 1 mm (20% of a specimen height) and a 1 Hz pre-conditioning cycle.^14, 37^ A sinusoidally varying displacement was then performed with 1% dynamic amplitude between 0.95 and 1.05 mm (i.e. from peak to trough) across a frequency sweep of 0.5–35 Hz. This frequency range is relevant to the strain rates comparable with previous studies on porcine^40^ and human brain tissue,^19^ and to which the brain might be exposed during physiological and traumatic loading conditions.^26^ For each frequency, the sinusoidal force and displacement data were recorded and analyzed using a Fast Fourier Transform (FFT). The data-set length for force (F*) and displacement (d*) at the fundamental frequency were quantified and used to calculate the dynamic stiffness (k*). Then, the storage (*E*′) and loss (*E*″) moduli were calculated by converting from the relevant stiffness through a shape factor from:

1 $$\begin{array}{c}{k}^{\ast}=\frac{{F}^{\ast}}{{d}^{\ast}}\end{array}$$

2 $$\begin{array}{c}{E}^{\prime}=\frac{{k}^{\ast}{\text{cos}}\delta }{S}\end{array}$$

3 $$\begin{array}{c}{E}^{\prime\prime}=\frac{{k}^{\ast}{\text{sin}}\delta }{S}\end{array}$$

4 $$\begin{array}{c}S=\frac{\pi {d}^{2}}{4h}\end{array}$$

where *h* and *d* are the thickness and diameter of a specimen. The phase angle $\delta$ is the phase lag between the applied compressive force and displacement. *S* is the shape factor for cylindrical samples. Further details on the characterization are provided elsewhere.^52^

For the stress relaxation tests, specimens were subjected to a compressive strain of 0.1 and a relaxation step of 150 s was followed at this compression level. The process of stress relaxation shows how the stress induced in the material reduces following sudden deformation, from the corresponding stress-strain data and material’s viscoelastic response can be evaluated. The velocity of 180 mm/min was set for the compression step. The stress was calculated from the ratio of measured force and sample original cross-sectional area.

**Figure 2:**
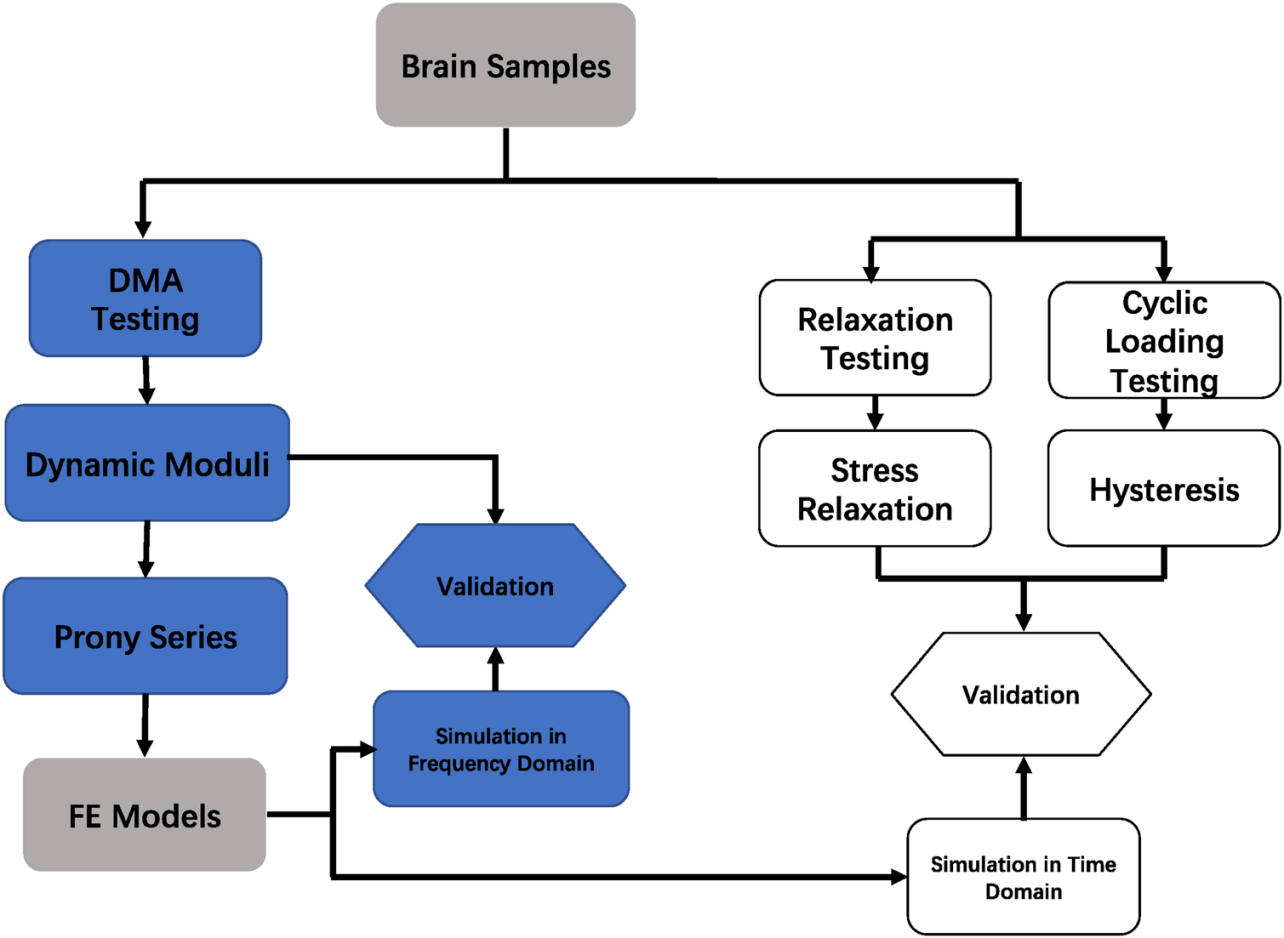
Outline of the experimental design used in this study. Blue boxes denote workflow linked to frequency domain and white boxes denote the workflow linked to time domain.

Further, samples were subjected to a sinusoidal compression with cyclic loading at a frequency of 35 Hz with 0.05 mm dynamic amplitude for about 2 s to collect hysteresis loops. A lag between the unloading and loading portions of the curve exist for a viscoelastic material. A total of 55 white matter and 41 grey matter samples were tested in the frequency domain through DMA, and 8 white matter and 10 grey matter samples were tested through stress relaxation and cyclic loading measurements. All 114 test samples were tested at room temperature and hydrated with Ringer’s solution during the testing. The collected experimental data were initially used to determine the viscoelastic parameters (from frequency domain tests) and compared with FE models under both testing domains for validation. For clarity, a schematic showing the experimental design is outlined in Fig. 2.

Sigmaplot version 14.5 (Systat Software Inc., London, UK) was used for statistical analysis. Two-way analysis of variance (ANOVA) was performed for all pairwise comparisons between brain regions with Tukey post-hoc analysis. Statistical tests were assumed to be significant at 5% level.

### Constitutive Modelling

**Figure 3:**
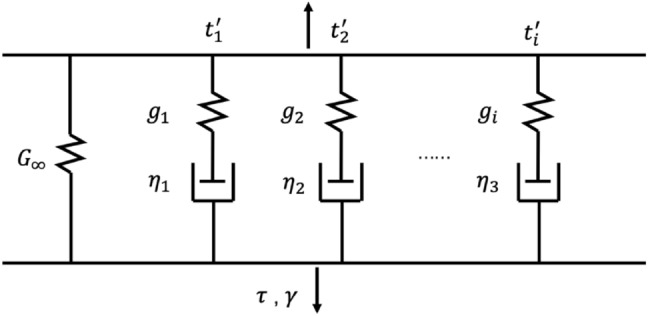
A schematic interpretation of the generalized Maxwell model.

Linear viscoelastic theory has been used in computational studies to analyze the patterns of brain injuries and the relationship between strain and stress.^9, 13^ In addition, this model can be effectively applied in commercial FE software. The time dependent response of the material is applied in the model determining the stress relaxation ($\tau \left(t\right)$) for a viscoelastic model:

5 $$\begin{array}{c}\tau \left(t\right)={\int }_{0}^{t}\mu \left(t-{t}^{^{\prime}}\right)\dot{\gamma }\left({t}^{^{\prime}}\right){\text{d}}{t}^{^{\prime}}\end{array}$$

where $\dot{\gamma }$ is the strain rate tensor and $\mu (t)$ is the time-dependent relaxation modulus. The generalized Maxwell model is widely used to characterize the modulus function for linear viscoelastic materials with a main elastic branch and *N* spring-dashpot pair branch shown in Fig. 3. Using the Prony series, the constitutive relation of the viscoelastic response in the time domain is as follows:

6 $$\begin{array}{c}\mu \left(t\right)={G}_{\infty }+\sum_{i=1}^{N}{g}_{i}\mathrm{exp}\left(-\frac{t}{{t}_{i}^{\prime}}\right)\end{array}$$

where ${G}_{\infty }$ is the equilibrium modulus, ${g}_{i}$ and ${t}_{i}^{\prime}$ are the relative moduli and the relaxation time of the Prony series for *N* relaxation modes where ${t}_{i}^{\prime}={\eta }_{i}/{g}_{i}$; ${\eta }_{i}$ is the corresponding viscosity. The initial stress modulus can be obtained from the sum of ${G}_{\infty }$ and ${g}_{i}$.

In the frequency domain, Eq. (5) can be transferred to the Laplace form by considering a pure imaginary variable *s* to $j\omega$ as:

7 $${u}^{\ast}(j\omega)= s{\tilde{u}}(s)=\frac{{\tilde{\tau}}(s)}{{\tilde{\gamma}}(s)}=\frac{{\tilde{\tau}}(j\omega)}{{\tilde{\gamma}}(j\omega)}$$

where $\tilde{u }$ is the relaxation modulus, $\stackrel{\sim }{\tau }$ is the stress tensor and $\stackrel{\sim }{\gamma }$ is the strain tensor in the Laplace form. $\omega$ is an angular frequency and $j= \sqrt{-1}$. The complex modulus ${u}^{\ast}$ can be expressed from the dynamic storage modulus ${u}^{\prime}$ and loss modulus ${u}^{\prime\prime}$ as:

8 $$\begin{array}{c}{u}^{\ast}={u}^{\prime}+j{u}^{\prime\prime}\end{array}$$

A discrete relaxation spectrum is considered in physical models. The relaxation modulus $\mu \left(t\right)$ expressed above is in the form of a discrete set of exponential decays. Using this discrete function, the complex modulus ${u}^{\ast}$ can then be defined as:

9 $$\begin{array}{c}{u}^{\ast}\left(j\omega \right)={G}_{\infty }+\sum_{i=1}^{N}{g}_{i}\frac{{t}_{i}^{\prime}j\omega }{1+{t}_{i}^{\prime}j\omega }\end{array}$$

Thus, the Prony series representations of dynamic storage and loss modulus in the generalized Maxwell model can be obtained as functions of frequency:

10 $$\begin{array}{c}{u}^{\prime}\left(j\omega \right)={G}_{\infty }+\sum_{i=1}^{N}{g}_{i}\frac{{{(t}_{i}^{\prime}\omega )}^{2}}{1+{{(t}_{i}^{\prime}\omega )}^{2}}\end{array}$$

11 $$\begin{array}{c}{u}^{\prime\prime}\left(j\omega \right)=\sum_{i=1}^{N}{g}_{i}\frac{{t}_{i}^{\prime}\omega }{1+{{(t}_{i}^{\prime}\omega )}^{2}}\end{array}$$

The dynamic modulus and relaxation modulus shared the coefficients. The parameters of discrete frequency dependent relaxation modulus are estimated using a non-linear least algorithm by calibrating the constitutive models with the average experimental data based on the average square of deviation between the measured dynamic modulus from the mechanical tests and the predicted values *via* Eq. (12).

12 $$\begin{array}{c}\sum_{j=1}^{m}\left({\left(\frac{{u}^{\prime}\left({\omega }_{j}\right)}{{\overline{u} }_{j}^{\prime}}-1\right)}^{2}+{\left(\frac{{u}^{\prime\prime}\left({\omega }_{j}\right)}{{\overline{u} }_{j}^{\prime\prime}}-1\right)}^{2}\right)={\text{m}}{\text{i}}{\text{n}}\end{array}$$

where ${\overline{u} }_{j}^{\prime}$, ${\overline{u} }_{j}^{\prime\prime}$ are the measured dynamic modulus at *m* frequencies ${\omega }_{j}$ with ${u}^{\prime}\left({\omega }_{j}\right)$ and ${u}^{\prime\prime}\left({\omega }_{j}\right)$ the predicted values calculated from Eqs. (10) and (11), respectively. From here, the relaxation times ${t}_{i}^{\prime}$ are expected to be prescribed and the coefficients ${g}_{i}$ are subsequently calculated. The resulting constants are considered all positive. The spacing of relaxation times has been suggested around 1 logarithmic time^39^ and negative coefficients may appear when the interval is too small.^20^ In addition, the number *N* of Maxwell elements is an important issue for the success of the nonlinear method. A large number of relaxation modes generally leads to higher accuracy, but more complexity is generated and negative constants start to occur with ill-posed issues.^45^ In this study, the initial number of relaxation elements was empirically chosen around ten for transmission and redundant elements can be merged or eliminated. From the preliminary studies, an eight term Prony series was chosen for these linear viscoelastic models. The goodness of fit of data to the given model was assessed using the coefficient of determination *R*^2^.

### FE Simulations

**Figure 4:**
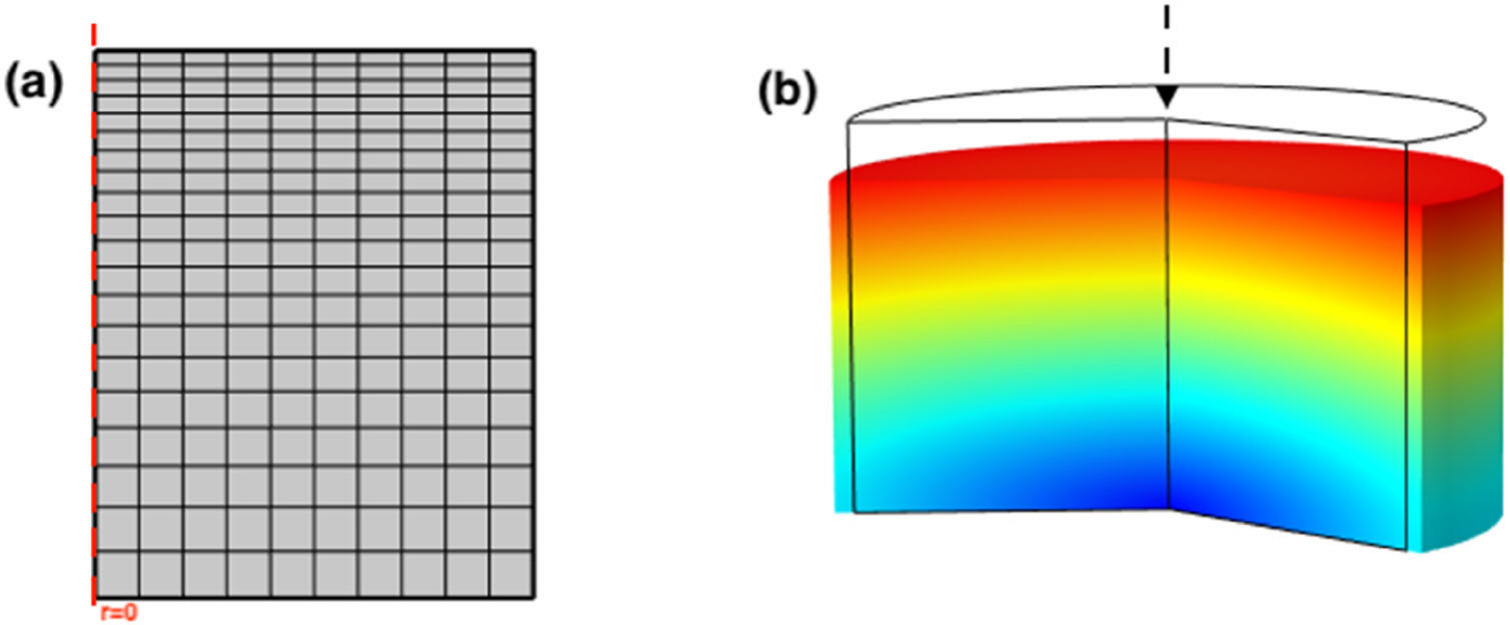
Finite element simulation used for the uniaxial compression of brain tissue in (a) axisymmetric and (b) deformed 2D revolution configurations.

The average mechanical behavior of brain tissue for white and grey matter was simulated, separately, both in the frequency and time domain using COMSOL Multiphysics 5.5 (COMSOL, Stockholm, Sweden). For simulations in both domains, an axisymmetric model was used with a cylindrical geometry representing the average tested brain specimen of 4 mm in radius and 5 mm in thickness. The bottom surface was restrained vertically while it was free to move and expand horizontally. A linear viscoelastic model was applied under the COMSOL solid mechanics module. To avoid ill-conditioning for incompressible materials in the FE simulation, a Poisson’s ratio of 0.49 was chosen.^34^ The viscoelastic parameters obtained from dynamic modulus of white and grey brain tissue (derived from “ Constitutive Modelling” section) were inputted into the general Maxwell material constitutive model with eight viscoelastic branches under material setting to represent the mechanical behavior for both testing methods. A mapped 4 node was employed for brain tissue to create an axisymmetric quadrilateral mesh on boundaries and an element ratio node was applied to specify the element size in the distribution (Fig. 4). The applied mesh density was validated by a mesh convergence analysis.

In the frequency domain analysis, the top surface was displaced on brain tissue by 1 mm in the vertical direction, followed by a harmonic perturbation of 0.05 mm over a range of frequencies, 0.5–35 Hz. These models were solved under conditions which mimicked the experimental conditions of DMA tests.

In the time domain analysis, the brain tissue was compressed to 0.1 strain and held for a relaxation step consistent to the experimental conditions to obtain simulated stress relaxation results. In addition, a sinusoidal prescribed displacement was set under the time dependent solver at 35 Hz for 2 s in the form $\mathrm{sin}\left(2\pi ft\right)$, where *f* is the testing frequency and *t* is the time, to obtain the force displacement viscoelastic hysteresis loops. The comparison between the FE models and physical tests were used for validation.

## Results

### Frequency Dependency of Viscoelasticity

**Figure 5:**
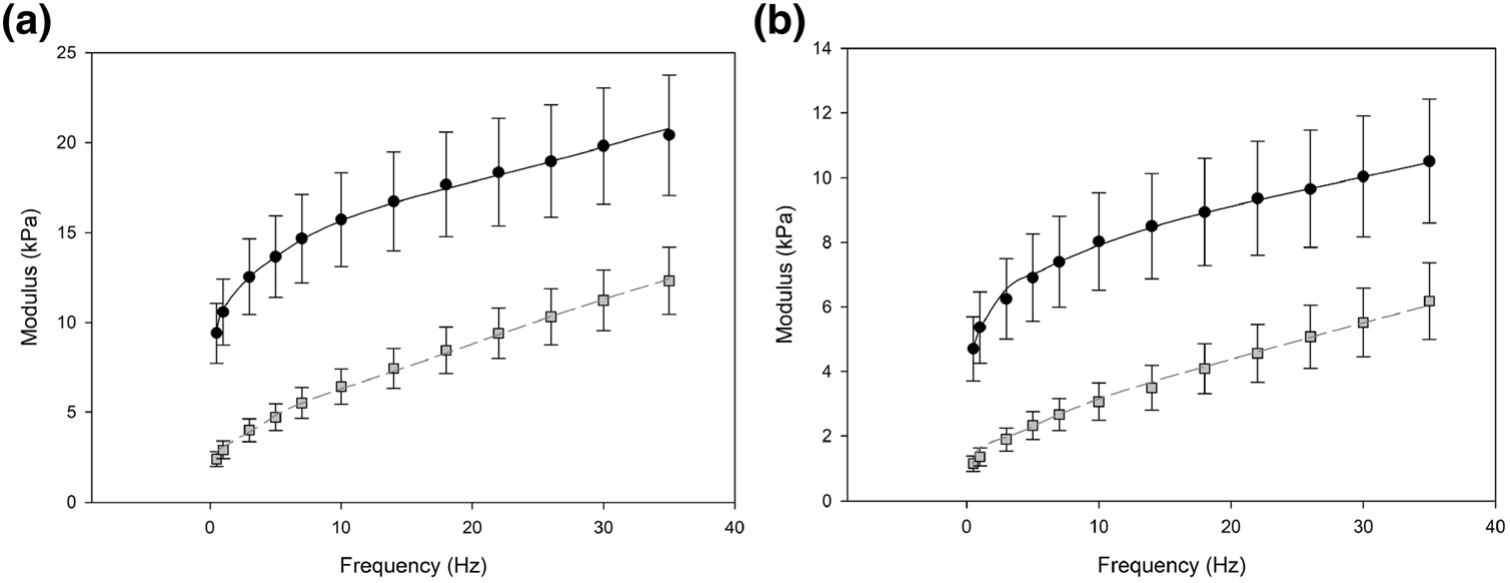
Variation of mean storage (circle) and loss (squares) moduli against frequency for (a) white and (b) grey matter tissue obtained using DMA. Error bars represent 95% confidence intervals. Predictions of dynamic properties from FE simulations, in the frequency domain, are shown as the lines for dynamic storage (full black line) and loss (dashed grey line) modulus.

The frequency dependent mechanical behaviors of brain white and grey matter were characterized through dynamic mechanical testing, and our results show that the storage modulus is greater than the loss modulus over all tested frequencies. Figure 5a illustrates an increasing trend with frequency for white matter storage and loss modulus with average values of 15.72 and 7.97 kPa, respectively. Figure 5b illustrates the significantly lower storage and loss modulus (*p* < 0.05) for grey matter with average values of 7.97 and 3.45 kPa, respectively. The mean results of the experimental dynamic moduli of brain tissue tested from various regions were used to determine the optimized parameters of the discrete relaxation spectrum with eight term Prony series (Table 1) with fairly good fitting results (0.996 < *R*^2^ < 0.999) and the equilibrium modulus was 0.48 kPa. The number of eight pairs of relaxation modes was adequate to simulate the mechanical behavior converted from the frequency-domain and redundant modes were merged. The FE models in the frequency domain were capable of capturing the trend for both storage and loss moduli across the frequencies investigated.

**Table 1:** Material parameters of relaxation moduli obtained from the mean dynamic viscoelastic properties for white and grey brain matter.

	Linear viscoelastic model parameter			
	White matter		Grey matter	
$i$	${g}_{i} \left({\text{kPa}}\right)$	${t}_{i}^{\prime} \left({\text{s}}\right)$	${g}_{i} \left({\text{kPa}}\right)$	${t}_{i}^{\prime} ({\text{s}})$
1	24.33	7.36 × 10^−4^	7.30	1.00 × 10^−4^
2	19.37	2.23 × 10^−3^	17.88	1.45 × 10^−3^
3	4.87	2.51 × 10^−2^	2.43	1.55 × 10^−2^
4	4.46	2.73 × 10^−1^	2.43	1.45 × 10^−1^
5	2.43	1.00 × 10^1^	1.46	1.00 × 10^1^
6	2.43	1.00 × 10^2^	1.46	1.00 × 10^2^
7	1.46	1.00 × 10^3^	0.49	1.00 × 10^3^
8	0.49	1.00 × 10^4^	0.29	1.00 × 10^4^

### Time Dependency of Viscoelasticity

**Figure 6:**
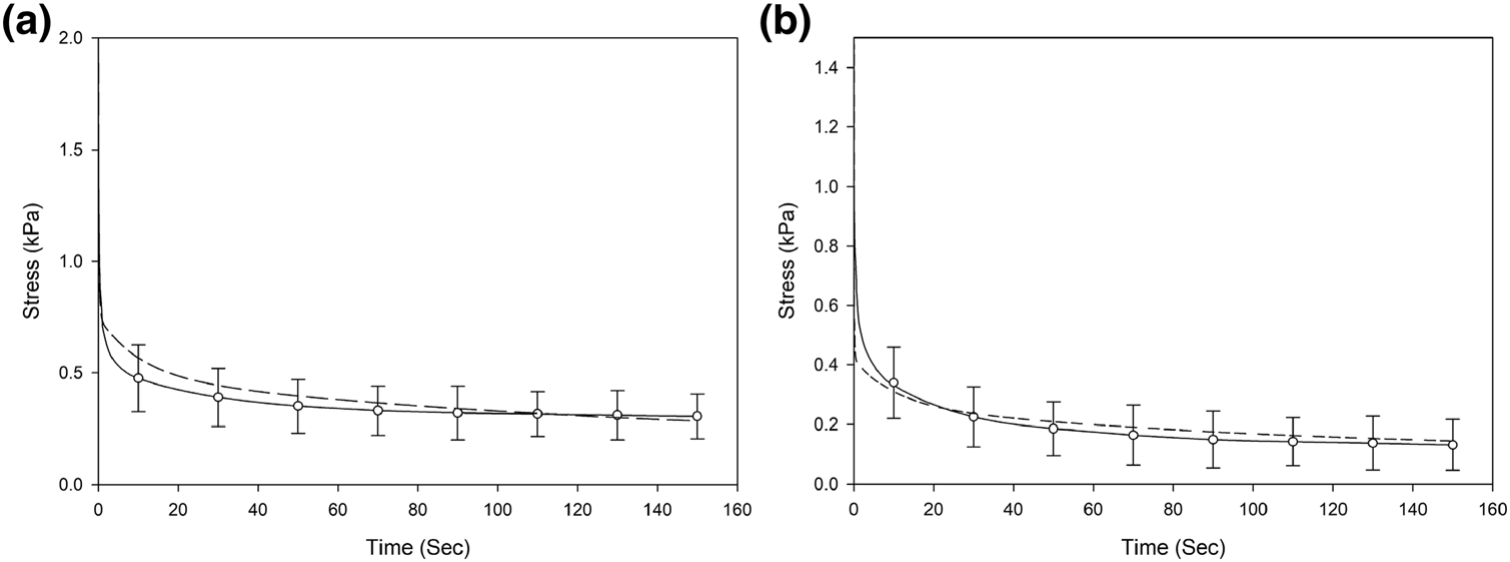
Relaxation response of (a) white and (b) grey matter tissue obtained from the stress relaxation tests (full line) with 95% confidence intervals shown as error bars, and the prediction of stress relaxation (dash line) based on frequency domain derived parameters from FE simulations in the time domain.

The mean stress relaxation behaviors were obtained and the material relaxation for both white and grey matter (Fig. 6) showed immediately a drop after the compression platen was held. The stress drop for white matter is higher than that of grey matter. The viscoelastic parameters converted from dynamic modulus were applied in the time dependent simulations and the models were able to approximate the trend of stress relaxation responses. For the white matter, the prediction of the stress relaxed slower at the beginning than the experimental results, followed by a faster relaxation and with longer relaxation process, the viscoelastic response was more closely approximated with a difference of less than 19%. For the grey matter, the prediction results appeared to relax faster at first and then exhibited similar relaxation behavior with a difference of up to 13%. The viscoelastic response in prediction of models was mostly approximated within the 95% confidence intervals through the measured relaxation process.

**Figure 7:**
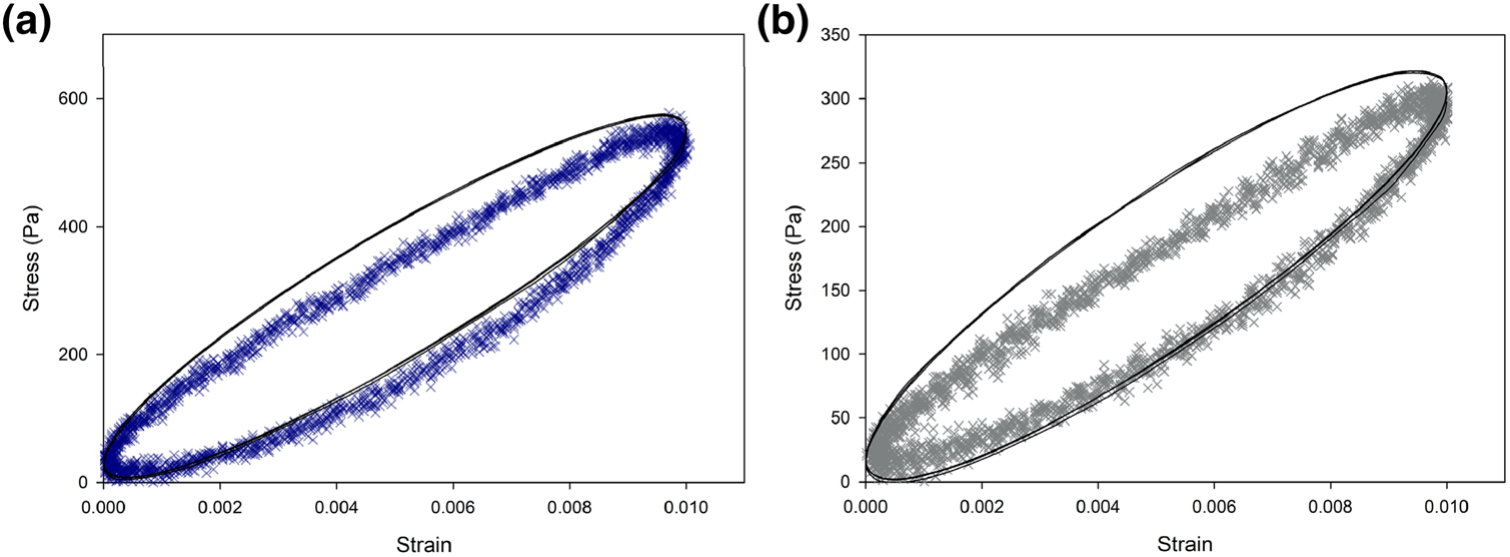
Representative hysteresis loops of stress against strain for (a) white matter and (b) grey matter tissue obtained from cyclic loading tests with model prediction results (black line) based on frequency domain derived parameters following FE simulations in the time analysis.

The hysteresis loops for white and grey matter tissue are shown in Fig. 7 as a measure of observing the dissipated energy for the material. Under the same testing protocols, samples from white matter exhibited a larger hysteresis area than samples from grey matter, meaning the greater amount of energy dissipated for white matter tissue. The curves for both white and grey matter were approximately elliptical. This indicated the tested specimens showed linear viscoelastic mechanical behavior. In simulations, the viscoelastic parameters converted from dynamic modulus were applied in the time dependent models and used to predict the stress strain relationship; the range of stress was estimated well for both white and grey matter. The hysteresis behavior for white matter was closely approximated by model prediction with a difference in the area enclosed by hysteresis loops of up to 18%. For grey matter the predicted area was larger than the experimental results, with up to a 34% area difference.

## Discussion

This study has investigated the viscoelastic properties of brain tissue under time and frequency testing domains and computational models were performed to predict the mechanical behavior based on the parameters of a discrete relaxation spectrum from dynamic moduli. Dynamic mechanical experiments are effective for measuring the viscoelastic properties of biological tissue over a range of frequency and the dynamic properties of brain tissue measured can be converted in the time domain data which are applicable in engineering analysis. Frequency-dependent storage and loss moduli were collected from brain white and grey matter tissue in compression. Stress relaxation tests were performed to obtain the time-dependent viscoelastic behaviour and brain samples were subjected to a sinusoidal compressive displacement in the time domain to obtain the hysteresis loops. For different testing protocols, the brain samples were kept under the same physical conditions. The time-dependent experimental results were compared to the predictions from simulations based on the constitutive linear viscoelastic, converting frequency to time-domain data. This is the first study to validate the use of viscoelastic data of brain tissue, derived from the frequency-domain, for use in FE models in the time-domain.

The dynamic mechanical properties of brain tissue showed an increasing trend over tested frequencies from DMA measurements, which is in agreement with previous studies on porcine brains^22^ as well as other biological materials, including human bladder tumors.^2^ Frequency-dependent dynamic moduli showed brain white matter is stiffer. A similar trend for the regional difference was found on human brain tissue.^24^ A wide range of loading conditions has been applied to determine the frequency-dependent viscoelastic properties. Human brain tissue has been studied in shear^17^ and the frequency-dependent behaviour of porcine bladder was characterized in tensile.^3^ Further, brain tissue has been investigated in the frequency domain through magnetic resonance elastography (MRE).^15^ However, the dynamic compressive properties of brain tissue have not been understood completely.^12^ The compressive force is important in the analysis of brain injuries where the brain could be exposed to compressive waves during the course of head impact.^36^ Even though potential differences exist in the various testing methods compared, the general trends for the dynamic storage and loss modulus vs frequency on these biological tissues were consistent across these.

The standard mechanical models have been applied in this study to capture the linear viscoelastic material functions of brain tissue with Prony series representations, and the fitting of dynamic moduli from experiments and the time domain material functions are subsequently obtained. There are other mathematical models available to describe the linear viscoelastic response of a material.^47^ The fractional derivative models with a fractional order ‘spring-dashpot’ element were used to determine the viscoelastic behavior in relaxation tests on brain tissue.^57^ The modified power law, derived from the phenomenology of polymer was applied on soft and biological materials to describe their power law viscoelastic response from a wide range of test conditions.^7^ These models are able to characterize viscoelastic properties using model coefficients, the determination of the constants from experimental results generally is less efficient due to the complicated mathematical expressions and it is limited for the conversion of parameters across material functions.^38^

The parameters in the simulations for linear viscoelastic models were determined by fitting the dynamic storage and loss modulus from experiments. There are various techniques for the fitting described in the literature. A simple collocation method has been applied on polymethylmethacrylate to fit the viscoelastic behavior from dynamic shear and tensile relaxation tests.^41^ A least squares method was widely used to obtain model coefficients on brain tissue viscoelastic properties and has the benefits of being easily implemented in commercial software.^8, 13^ In this study, the frequency dependent responses of brain white and grey matter tissue were evident from the discrete relaxation mode with the exponentially ascending order of relaxation times and positive constrains, which is consistent to a previous study which analyzed dynamic mechanical data.^5^ The technique presented in this study can have wider applications for other biological tissues such as coronary arteries^10^ and mitral valve,^52^ where the frequency dependent properties have been investigated and described using simple fitting equations obtained through regression analysis. It enables the viscoelastic properties of the brain to be measured under realistic, dynamic conditions and makes this information available for brain models which predict trauma.^33, 46, 53^

The viscoelastic characterization of brain tissue in the time domain was studied through stress relaxation tests and the hysteresis loops were obtained to characterize the dissipated energy. The experimental results show that the behavior of brain tissue is not only frequency-dependent but also time-dependent. Further, the viscous relaxation for white and grey matter was similar with a stress relaxation of around 85% which is in agreement with a previous study on human brain tissue.^8^ Hysteresis loops for white matter showed a larger area than that for grey matter. A similar trend was found on bovine brain tissue indicating that the white matter with larger dissipated energy shows more viscous than grey matter.^49^

In FE simulations, the models with linear viscoelasticity were able to accurately capture the dynamic storage and loss modulus for both brain white and grey matter in the frequency domain. The model viscoelastic parameters were collected from dynamic mechanical tests and time domain material functions were derived based on the Prony series representation. Although the mild brain traumatic loading conditions were the focus of this study, there is future opportunity to investigate the applicability of the model at higher loading rates, such as blast brain impacts.^48, 42, 51^ A previous study investigated the differences of converting dynamic modulus to relaxation modulus, however, there was no direct experimental data for validation.^56^ The simulated results of the time domain in this study showed the general trends for stress relaxation behaviour on brain tissue which is comparable to the experimental data. Despite the initial difference between the predicted and measured results, the viscoelastic response in prediction of models was mostly approximated within the 95% confidence intervals which indicated the prediction of models was considered reliable. The hysteresis area for both brain white and grey matter was predicted to be larger in simulations and the simulated hysteresis area for white matter was larger than that of grey matter which is consistent to the experimental trends. The approach presented in this study of converting material properties between frequency and time domains enables brain modelling in the time domain based on the mechanics of brain tissue measured under dynamic loading conditions.

To conclude, the viscoelastic behaviour of brain tissue was investigated under both time and frequency domains. Frequency-dependent storage and loss moduli were collected for both white and grey matter through dynamic mechanical tests which can be represented accurately by the linear viscoelastic models. The time-domain material functions were obtained through the corresponding frequency-domain material functions based on a Prony series representation. The stress relaxation and hysteresis characterizations were studied and compared to the predictions from model simulations. The outcomes provide a better understanding of the material viscoelastic behaviour and the linear viscoelasticity between the time and frequency dependent material functions of biological tissues. This analysis is of importance for a number of applications, for brain tissue is enables various loading conditions to be simulated using finite element simulations, including traumatic loading.
